# Reliability of 3D image analysis and influence of contrast medium administration on measurement of Hounsfield unit values of the proximal femur

**DOI:** 10.1371/journal.pone.0241012

**Published:** 2020-10-21

**Authors:** Hye-Won Lee, Hong Il Ha, Sun-Young Park, Hyun Kyung Lim

**Affiliations:** 1 Department of Radiology, Hallym University Sacred Heart Hospital, Anyang-si, Gyeonggi-do, Republic of Korea; 2 Department of Radiology, Soonchunhyang University Seoul Hospital, Seoul, Republic of Korea; University of Groningen, University Medical Center Groningen, NETHERLANDS

## Abstract

**Purpose:**

To evaluate the reliability of 3D image analysis and the effect of an iodine contrast agent on the computed tomography (CT) Hounsfield unit (HU) values of the proximal femur.

**Materials and methods:**

Fifty female patients (mean age, 61.3 years; age range, 50–79 years) who underwent both pre- and post-enhancement abdominopelvic CT scans were included in this retrospective study. Whole 3D volumes of the left proximal femur from the head to the lesser trochanter were extracted using the regional growth technique with commercial 3D software. Total volume, mean HU, and HU histogram analysis (HUHA) values of the extracted femur were calculated. HUHA distribution was classified into HUHA_fat_ for the assumed fatty marrow (percentage of negative HU values) and HUHA_dense-bone_ (percentage of HU values ≥ 126 HU). Reliability was assessed by calculating intra- and interobserver correlation coefficients (ICCs) and by drawing Bland–Altman plots. The effect of contrast medium administration was evaluated by the paired *t*-test.

**Results:**

All intra- and interobserver ICCs of 3D volume measurements showed excellent reproducibility (all ICCs > 0.90). On Bland–Altman analysis of two observers’ 3D volume measurements, the differences in the mean total volume, HUHA_fat_, HUHA_dense-bone_, and mean HU were 2.4 cm^3^, 0.17%, 0.6%, and 1.9 HU, respectively. The mean difference in HU after contrast agent administration (-2.2 HU) was not significant (*P* = 0.27). The mean difference in HUHA_fat_ and HUHA_dense-bone_ after contrast agent administration were -1.1% and -2.2%, respectively, on the Bland–Altman plot. HUHA_fat_ and HUHA_dense-bone_ showed significant differences (*P* < 0.05). The 95% limits of agreement for HUHA_fat_, HUHA_dense-bone_, and mean HU were -3.6% to 1.3%, -6.5% to 2.1%, and -30.0 to 25.5 HU, respectively.

**Conclusion:**

Image analysis based on 3D volume measurement of the proximal femur showed excellent reliability, with the contrast agent administration showing negligible influence on the mean HU.

## Introduction

The major advantage of imaging analysis using computed tomography (CT) is its reliability [1–3]. Quantification of CT data is usually performed on the measurement of mean HU values. Recent advances in three-dimensional (3D) image analysis software have made it possible to draw HU histograms of regions of interest (ROIs) [4], which can be subclassified by HU range to calculate the proportions of fat and cortical bone. This analysis shows a strong positive correlation and high diagnostic performance for predicting osteoporosis of the femoral neck in comparison with analyses based on the mean HU value [5]. However, ROI-based image analysis has two major disadvantages. First, observer subjectivity cannot be totally excluded when drawing an ROI. Second, if the target organ has three-dimensional internal structure complexity, as in the case of the proximal femur, HU measurements may be under- or overestimated depending on the selected ROI [5]. The latest medical image analysis techniques allow extraction of bone from CT images and calculate the mean HU value and selected range of the HU histogram distribution for a specific volume of interest (VOI) [6–9]. VOI-based HU measurement can be an alternative to ROI-based measurement because this analysis technique can not only reduce observer prejudice in image selection but also eliminate the effect of the three-dimensional complexity of the target lesion. However, reliability evaluation of this technique has not yet been reported.

In the vertebral body, where blood vessels are abundantly distributed, the CT HU value increases after administration of the contrast agent. Thus, the mean HU value of the lumbar vertebral body is affected by the use of iodine contrast agents [10, 11]. Since the femoral head and neck contain few blood vessels and the femoral blood flow is low and decreases significantly with age [12], the femoral head and neck might show no or insignificant differences in the CT HU values measured before and after contrast agent administration. Moreover, precontrast CT scans are being increasingly omitted from routine CT scan protocols to reduce radiation exposure. Thus, if contrast agents have no effect on the CT HU measurement, the femoral head and neck can be an optimized target organ for evaluating bone density using CT, unlike the vertebral body. In addition, the femoral head and neck is considered the ideal site to assess osteoporosis because these structures are less affected by degenerative arthritis [13] and consist mainly of dense trabecular bone and Ward’s triangle [14, 15]. However, no previous study has assessed the changes in each HU range as well as the mean HU of the proximal femur after iodine contrast agent administration.

Thus, we evaluated the reliability of proximal femur extraction using commercial 3D VOI image analysis software and the influence of iodine contrast agent administration on the HU values of the proximal femur.

## Materials and methods

### Patients and CT protocol

**Fig 1:**
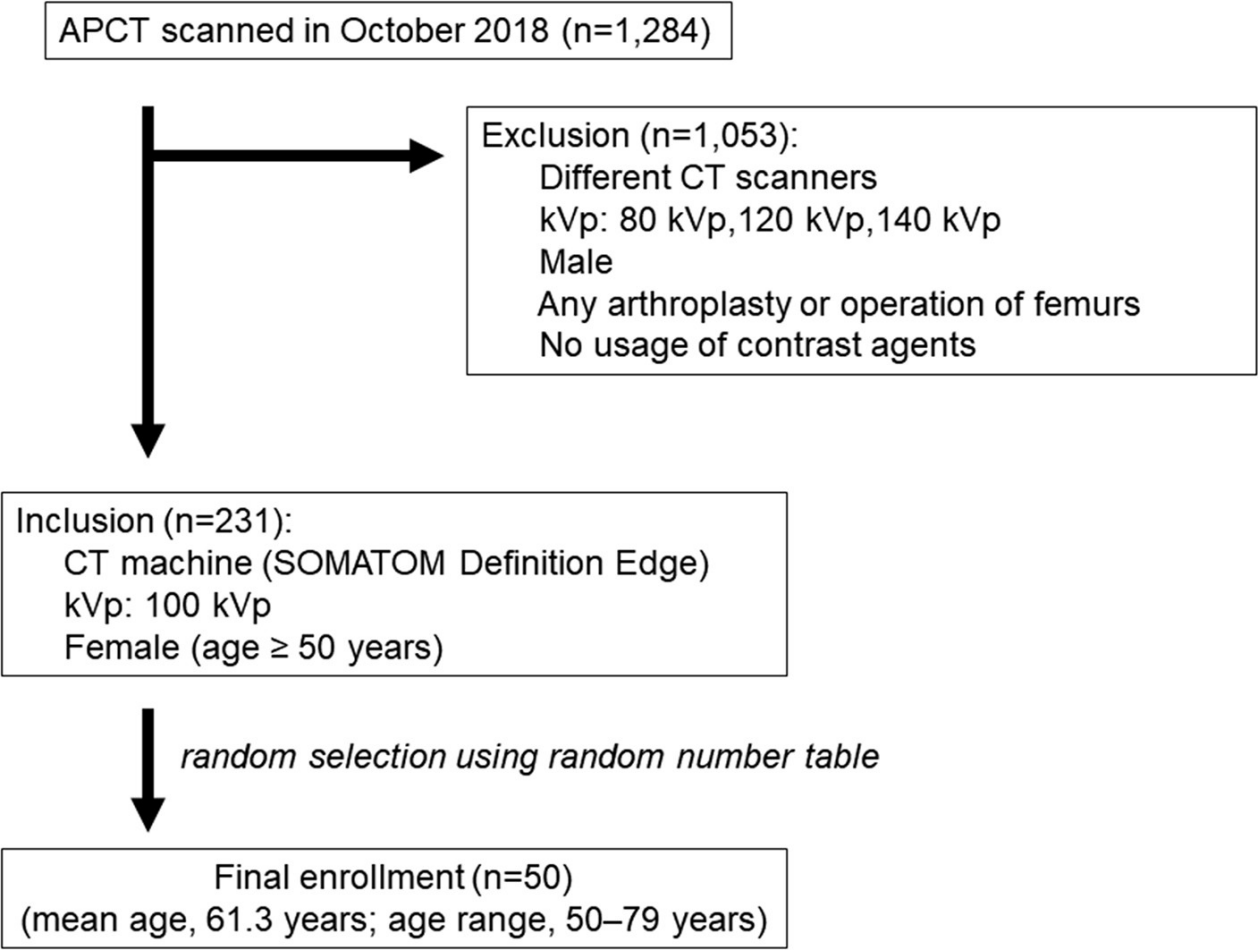
Flowchart of patient selection. APCT = abdominopelvic CT.

This retrospective study was approved by institutional review board and ethics committee at Hallym University Sacred Heart hospital and the need for informed consent was waived. The sample size was chosen to allow detection of a significant mean difference between matched groups using the paired *t*-test. Forty-five patients were required to afford a power of 95% and an α-error of 0.05. Thus, 50 female patients (mean age, 61.3 years; age range, 50–79 years) who underwent both pre- and postcontrast enhancement abdominopelvic CT (APCT) in October 2018 were randomly selected using a random number table, allowing for a 10% dropout caused by unexpected data loss (Fig 1). We excluded male patients and female patients with any bone disease at the femoral neck, any prosthesis in the femurs, or no use of contrast material. We included only female patients more than 50 years of age because osteoporosis is a major public health problem in the elderly female population. This reliability analysis was planned as a prior study for opportunistic screening of osteoporosis using APCT in a female cohort in our hospital. We only included APCT examinations performed using the same multidetector-row CT (MDCT) scanner (SOMATOM Definition Edge, Siemens Healthineers, Forchheim, Germany) operating in the standard single-energy CT mode. Automatic tube voltage selection (ATVS; Care kVp) and automatic tube current modulation (ATCM; CARE Dose 4D) were applied. Although ATVS includes kVp values from 80 to –140 kV, this study included only APCT scans taken at 100 kVp to exclude any effect of tube voltage. With each patient in the supine position, pre- and postcontrast enhancement APCT was performed from the diaphragm to the pubic symphysis. For each patient, 100–120 mL of a nonionic contrast agent (either iomeprol [Iomeron-300^®^, Bracco Imaging, Milan, Italy] or iohexol [Bonorex-300^®^, Central Medical Service, Seoul, Korea]) was injected at 3–5 mL/s using an automatic power injector; no additional saline was injected. Portal venous phase scans were obtained with a 70-s delay after the contrast material injection. No oral contrast agent was ingested. The scan parameters were as follows: detector collimation, 128 × 0.6 mm; pitch, 0.6; gantry rotation time, 0.5 s; tube current, 289 mA; tube voltage, 100 kVp; and application of sinogram-affirmed iterative reconstruction using a soft tissue kernel (I40f) at the S1 iteration level. The raw data were archived at a section thickness of 1 mm and an interval of 1 mm prior to 3D image analysis.

### Image analysis

**Fig 2:**
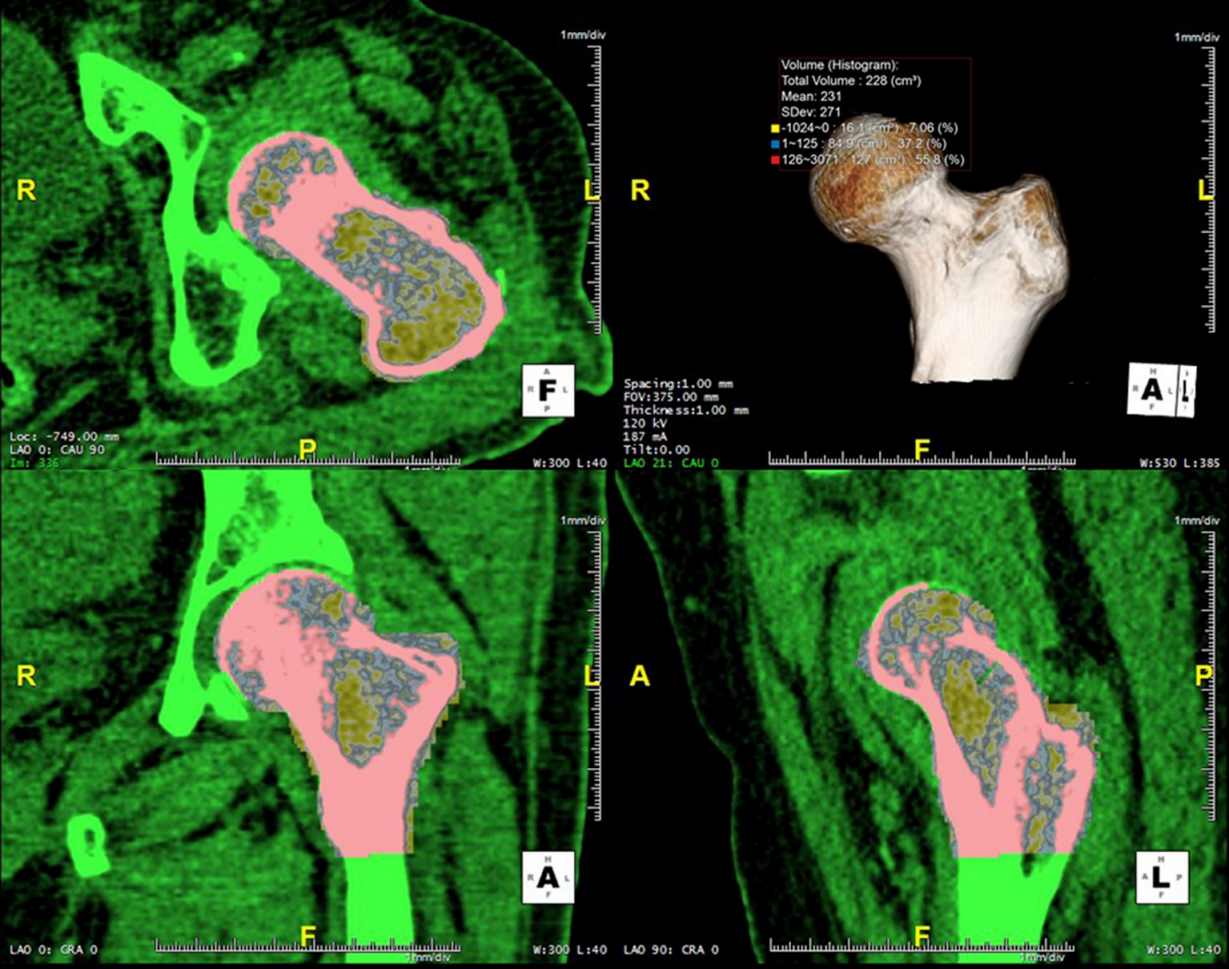
3D volume of interest (VOI) image analysis. 3D multiplanar reconstructions and volume-rendered image analysis were performed using commercial 3D software (Aquarius iNtuition v4.4.12^®^). The femur was selected on the volume-rendered image using the regional growth method by checking all 2D multiplanar reconstructed images. The entire bone of the proximal femur from the femoral head to the inferior margin of the lesser trochanter was extracted and analyzed. The total volume (cm^3^), mean HU (HU), and HU histogram analysis (HUHA) were automatically calculated. Each HUHA is shown as both a volume (cm^3^) and as a percentage of the VOI (%). The HUHA_fat_ (yellow box), which reflects the percentage of HU values of zero or less in the entire VOI, represents the fatty marrow. The HUHA_dense-bone_ (red box), which reflects the percentage of HU values of 126 or above in the entire VOI, represents dense cortical bone.

Image analysis was performed using commercial 3D software (Aquarius iNtuition v4.4.12^®^, TeraRecon, Foster city, CA, USA). The assessors did not undergo any specific interactive training sessions to learn the measurement techniques before conducting the measurements. The 3D VOI image analysis was performed in the following steps: first, the CT dataset was selected from the 3D image archive worklist, and the scan data were loaded into the 3D analysis software. Second, the left femur was selected and extracted using the regional growth method of the 3D volume-rendering protocol. The selected bone was identified in 2D images to confirm that all bone was included in the VOI. On the extracted 3D image of the femur, the observer drew a circle to select a VOI running from the femoral head to the inferior margin of the lesser trochanter. The total volume, mean HU, and HU histogram analysis (HUHA) values were automatically and simultaneously calculated (Fig 2). The HU value was measured from -1024 HU to 3071 HU, of which HU values of zero or less were marked as HUHA_fat_ and assumed to reflect fatty marrow (yellow box in Fig 2) and HU values of 126 or above were marked as HUHA_dense-bone_ and assumed to reflect cortical bone (red box in Fig 2). Each HUHA value was expressed as a percentage of the entire VOI (cm^3^) on the basis of a previous study [4]. Values obtained from pre- and postcontrast enhancement APCT images were tagged with the prefixes “Pre-” and “Post-,” respectively.

To assess the interobserver reliability, the volume extraction procedure was performed by two radiologists (first reviewer with 12 years of experience in interpreting abdominal images and a second reviewer with 6 years of experience in interpreting musculoskeletal images) with precontrast enhancement CT scans from 50 cases in a blinded manner. The intraobserver reliability was estimated by the first reviewer at a 4-week interval to prevent recall bias. All measurements on post-enhancement image sets were performed by the first reviewer.

### Statistical analysis

The intra- and interobserver reliabilities of 3D VOI image analysis were assessed by calculating the two-way mixed effect model of intraclass correlation coefficient (ICC) with absolute agreement. The ICC, defined as the proportion of the total error not associated with measurement error, was calculated. ICC values of <0.50, 0.50–0.75, 0.76–0.90, and <0.90 signified poor, moderate, good, and excellent reliability, respectively [16]. A paired *t*-test with or without Welch’s test was used to compare the mean HU and the HUHA values with respect to normal or unequal distributions. Bland–Altman plots and their 95% limits of agreement were used to explore differences between the observers’ measurements and between the pre- and post-enhancement APCT data [16, 17]. All statistical analyses were performed using MedCalc Statistical Software version 19.1 (MedCalc Software bv, Ostend, Belgium; https://www.medcalc.org; 2019). For all studies, a *P*-value < 0.05 was considered to indicate a statistically significant difference.

## Results

**Fig 3:**
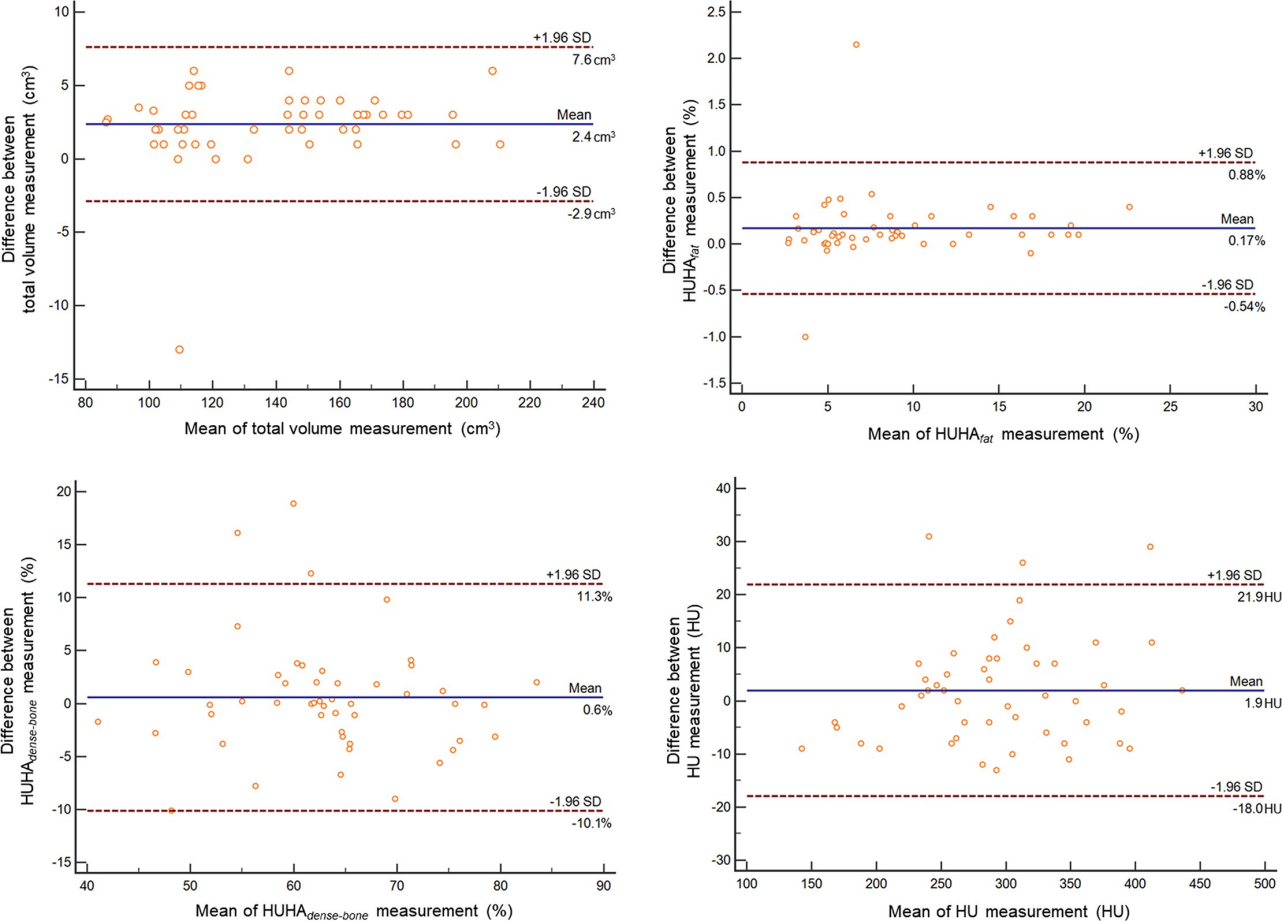
Bland–Altman plots of inter-rater agreement. Bland–Altman plots of measures of the total volume (A), the HUHA_fat_ (B), the HUHA_dense-bone_ (C), and the mean HU (D) show the relationships among measurements made by the two observers. Differences (y-axis) between duplicate measurements are plotted against the mean values (x-axis) of those measurements. Solid blue lines indicate mean differences. The top and bottom dashed lines correspond to the upper and lower margins of the 95% limits of agreement. At a probability of 95%, differences in normalized scores lie between the upper and lower limits of agreement (mean ± variability estimate = 1.96 standard deviations [SDs]).

Table 1 shows the intraobserver and interobserver reliability of the 3D VOI image analysis, with repeated measurements obtained on precontrast enhancement CT scans. This table shows excellent reliability (ICC > 0.9). In addition, there was no significant difference in the total volume on pre- and postcontrast enhancement CT scans (0.42 ± 6.52, *P* = 0.65). On the Bland–Altman plot analysis of interobserver measurements, the differences in mean total volume, HUHA_fat_, HUHA_dense-bone_, and HU were 2.4 cm^3^, 0.17%, 0.6%, and 1.9 HU, respectively (Fig 3).

**Table 1:** Intraclass correlation coefficients for reliability of the 3D VOI image analysis using a single-measure, absolute-agreement, and two-way mixed effect model.

	ICC	95% CI
Intraobserver agreement		
Total volume	0.9931	0.9646–0.9974
Mean HU	0.9949	0.9485−0.9984
HUHA_fat_	0.9988	0.9976−0.9993
HUHA_dense-bone_	0.9720	0.9508−0.9841
Interobserver agreement		
Total volume	0.9940	0.9642–0.9979
Mean HU	0.9882	0.9793−0.9933
HUHA_fat_	0.9971	0.9934−0.9985
HUHA_dense-bone_	0.9118	0.8449−0.9499

† CI, confidence interval; HU, Hounsfield unit; HUHA, Hounsfield unit histogram analysis; ICC, intraclass correlation coefficient.

**Fig 4:**
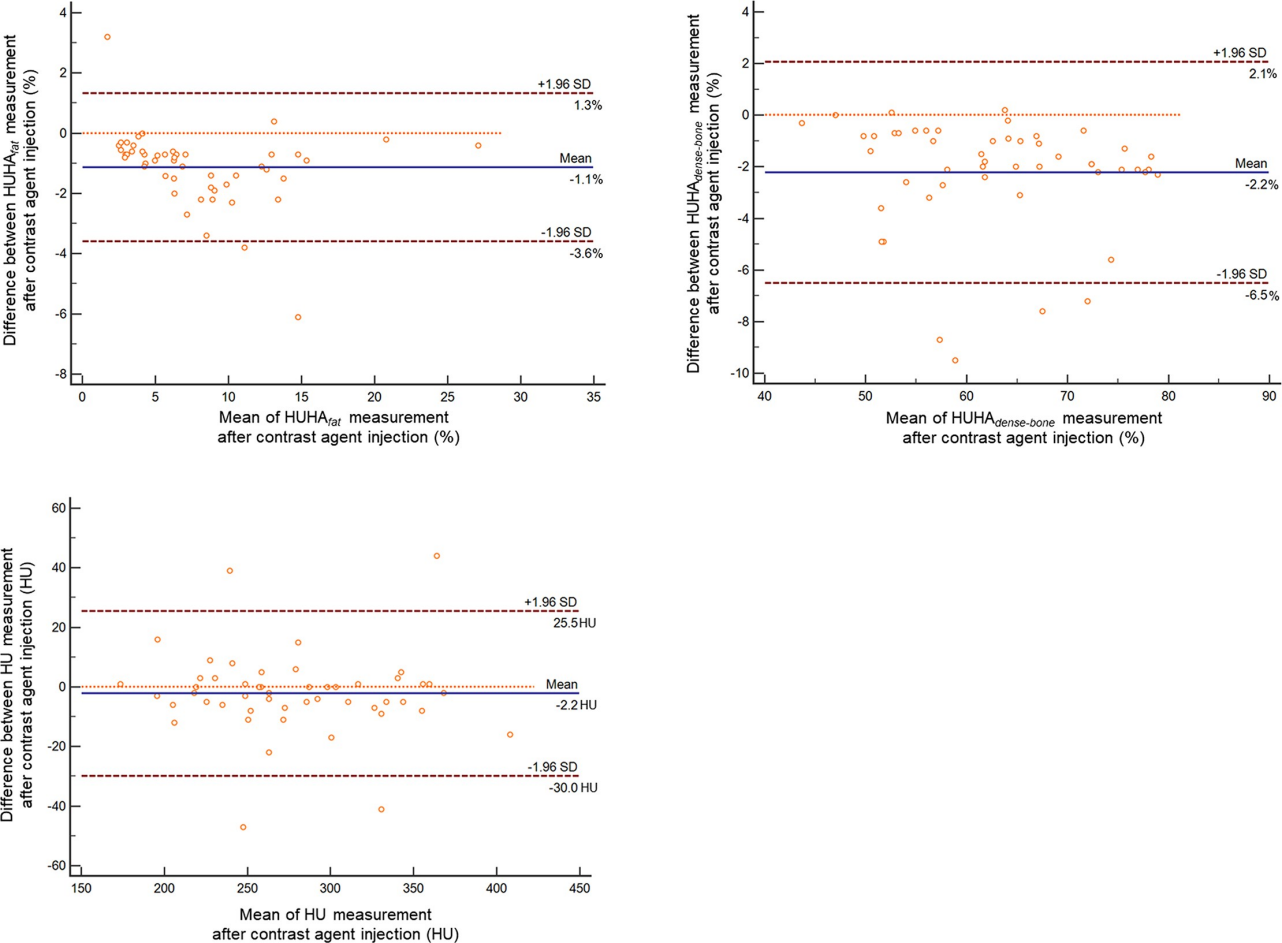
Bland–Altman comparisons of data derived from pre- and postcontrast enhancement CT scans. Bland–Altman plots for measurement of the HUHA_fat_ (A), the HUHA_dense-bone_ (B), and the mean HU (C) show mean differences with contrast agent administration. Differences (y-axis) are plotted against mean values (x-axis). Solid blue lines indicate mean differences. The top and bottom dashed lines are the upper and lower margins of the 95% limits of agreement. At a probability of 95%, differences in the normalized scores lie between the upper and lower limits (mean ± variability estimate = 1.96 standard deviations [SDs]).

Table 2 shows pairwise comparison results of the total volume, the mean HU, and the two HUHA values between the pre- and postcontrast enhancement CT scan datasets. The mean HU (2.2 HU) showed no significant difference (*P =* 0.27). The mean differences in the HUHA_fat_ and the HUHA_dense-bone_ after contrast medium administration were 1.1% and 2.2%, respectively. These values showed significant differences (P < 0.01). On the Bland–Altman analysis, the lower and upper 95% limits of agreement of the HUHA_fat_ were -3.6% and 1.3%, respectively, which were narrower than those of the HUHA_dense-bone_ (–6.5%, 2.1%) and the mean HU values (–30.0 HU, 25.5 HU) (Fig 4).

**Table 2:** Descriptions and pairwise comparisons of the mean HU, the HUHA_fat_, and the HUHA_dense-bone_ before and after contrast agent administration.

	Description of each value		Pairwise comparison		
	Pre-enhancement scan	Post-enhancement scan	Mean ± SD	95% CI	*P*-value^*^
	(mean ± SD)	(mean ± SD)			
Mean HU (HU)	276.82 ± 53.98	279.06 ± 54.70	2.24 ± 14.16	1.78–6.26	0.27
HUHA_fat_ (%)	8.53 ± 5.28	7.40 ± 4.94	1.13 ± 1.26	0.77–1.49	<0.01
HUHA_dense-bone_ (%)	61.55 ± 9.37	63.78 ± 9.59	2.23 ± 2.19	1.6–2.9	<0.01

* *P*-values were derived using the paired *t*-test followed by Welch’s test.

† C.I., confidence interval; HU, Hounsfield unit; HUHA, Hounsfield unit histogram analysis; SD, standard deviation.

## Discussion

In this study, 3D VOI image analysis based on measurement of HUHA_fat_, HUHA_dense-bone_, and mean HU values showed excellent agreement in both intra- and interobserver assessments. All mean differences in the HUHA, mean HU, and the total volume between the two observers’ measurement were less than 1%, 2.0 HU, and 2.5 cm^3^, respectively, in the Bland–Altman analysis.

Drawing the VOI and extracting the femur structure was the most subjective but important step in evaluating total volume measurement because it affected all results. Previous studies have reported high-level reliability of 3D imaging and semi-auto-segmentation analyses [3, 4, 18]. In particular, the trabecular bone of the proximal femur is asymmetric and anatomically complex in the 3D space, consisting of five trabecular groups, namely, the principal compressive, principal tensile, secondary compressive, secondary tensile, and greater trochanteric trabecula [19–21]. These groups form a complex internal structure, and it is impossible to accurately assess tissue composition using a single 2D image.

We had previously calculated the mean HU value and HUHA proportions on coronal reformatted images of the proximal femur [4] because we thought that the coronal reformatted image of the femur would be optimal, reflecting five trabecular groups on a single slice image, and thus better than axial and sagittal images when evaluating osteoporosis. We plotted the ROI on coronal reformatted images and included the largest area of Ward’s triangle, which is known as the most important structure for evaluating the osteoporotic femur [22, 15]. Although the ROI-based image analysis showed moderate to good reproducibility (kappa value, 0.67 to 0.86) in a previous study [4], the ROI selection itself was subjective since the process involved selection of one cross-section from several CT images, and subjective factors such as selection of the ROI location and determination of ROI size influenced the process of drawing the ROI on the selected image.

In contrast, although 3D VOI image analysis may still involve observer subjectivity, we standardized the analysis to minimize the observer’s intervention. The range of the target object was set by the index of the lesser trochanter inferior margin, and the proximal femoral volume was extracted semi-automatically using commercial 3D software. With this process, the 3D VOI image analysis showed excellent reproducibility in both intra- and interobserver ICC assessments. In addition to the advantage of excellent reproducibility, 3D VOI image analysis can be used to analyze the proportion of each variable in the whole proximal femoral volume rather than a specific area of the femoral head or neck. Therefore, 3D VOI image analysis would be a reasonable alternative to overcome the limitations of 2D ROI image analysis.

Pompe et al. and Pickhardt et al. used CT to study the HU value changes in lumbar vertebral bodies and reported that the mean differences were more than 11 HU after contrast medium administration. They reported that contrast medium administration substantially affected the mean HU value, and that the use of postcontrast enhancement CT scan data underestimated the extent of osteoporosis [10, 11]. Additionally, the recently published CT examination guidelines recommend that unnecessary precontrast enhancement CT scan should be refrained from in order to reduce radiation exposure [23]. This could be a potential limitation of APCT-derived opportunistic osteoporosis screening or bone density studies because the iodine contrast agent changes the HU value [24]. However, our study showed interesting results in this regard. After contrast agent administration, the mean HU difference in the proximal femur was less than 3 HU, and the difference was not significant. The HUHA_fat_ and HUHA_dense-bone_ values showed significant differences after the contrast agent administration on pairwise comparison; however, the mean difference of each value’s proportion was equal to or less than 2% in the Bland–Altman plot analysis. These contradictions between our findings and the results of the previous study may be explained by the measurement locations chosen in the two studies. Pompe et al. and Pickhardt et al. analyzed lumbar vertebral bodies, but we focused on the proximal femur. Each lumbar vertebral body has an abundant blood supply consisting of a pair of lumbar vertebral arteries branching from the aorta, and as each artery crosses a vertebral body, it gives rise to 10–20 ascending and descending branches termed primary periosteal arteries. The anterior spinal canal branch splits into the ascending and descending branches [25]. Moreover, the basivertebral vein is within the vertebral column contained in large, tortuous channels in the bone [26]. However, blood to the femoral head is supplied principally by the lateral and medial circumflex arteries, and the blood flow is low and decreases with age [15]. In addition, aging reduces blood flow and volume [27, 28]. Beam-hardening artifacts might be a possible contributing factor. The aorta is located just in front of the lumbar body, and the presence of a high level of contrast agent in the aorta might have affected measurements [24]. Here, we found that contrast agents negligibly affected HUHA_fat_, HUHA_dense-bone_, and the mean HU value in the proximal femur. Interestingly, the mean HU value did not differ significantly between pre- and postcontrast enhancement APCT scans, but the standard deviation was approximately 14 HU. The HUHA_fat_, on the other hand, showed a standard deviation of 1.3%. Further research is needed to establish cutoff values for applying these variables to the clinical practice of osteoporosis screening.

Our study had certain limitations. First, our study only included women older than 50 years. Age and gender bias are major limiting factors in clinical studies. Bone density itself is influenced by age and sex. However, our study was focused on evaluating the measurement reproducibility of each variable and the effect of contrast medium in the same patient. We thought that these two factors were not significantly affected by age or sex. Second, HUHA_fat_ and HUHA_dense-bone_, which represented fatty marrow and dense cortical bone, respectively, were arbitrary sets. Third, we used only one CT scanner. CT HU values may vary by CT manufacturer, CT model, and the convolution kernel used [29]. Thus, further validation with more CT scanners and settings is required. Fourth, we used two iodine contrast agents. However, this factor may not be of major concern because this study was analyzed by pairwise comparison. Fifth, our sample size was relatively small. This study designed paired data acquisition with 50 patients based on a two-tailed pre-hoc power analysis using an effect size of 0.5 and a prior statistical significance of 0.05. Thus, this study had a power of at least 93% [30].

In conclusion, 3D-VOI image analysis showed excellent reproducibility, while the changes in the mean HU measurements with contrast agent administration into the proximal femur were negligible.

## Abbreviations

APCT: abdominopelvic CT
HUHA: Hounsfield unit histogram analysis
ROI: region of interest
VOI: volume of interest
